# The antipsychotics functional index (AFI) in schizophrenia

**DOI:** 10.3389/fphar.2025.1591763

**Published:** 2025-07-02

**Authors:** Gabriel-Cristian Marinescu, Petru Iulian Ifteni, Andreea Teodorescu, Radu Georgescu

**Affiliations:** ^1^ Dr. Marinescu G Gabriel-Cristian CMI, Pitești, Romania; ^2^ Faculty of Medicine, Transilvania University of Braşov, Braşov, Romania; ^3^ Department of Psychiatry, University of Medicine and Pharmacy “Iuliu Haţieganu” Cluj-Napoca, Cluj-Napoca, Romania

**Keywords:** schizophrenia, antipsychotics, functionality, AFI, LAI

## Abstract

Schizophrenia can lead to significant and long-lasting deficits in patient functionality. The present study proposes a theoretical index that predicts the ability of an antipsychotic to improve the functionality of patients with schizophrenia. An advantage of this theoretical index is that it directly compares 29 first- and second-generation antipsychotics. This theoretical index, named the Antipsychotics Functional Index (AFI), was constructed considering factors such as pharmacodynamics, pharmacokinetics, pharmaceutical form, ease of administration, and safety aspects. A good antipsychotic ranking based on the proposed index results from combining the partial dopaminergic agonist mechanism and a lower frequency of administration. The top-ranked antipsychotic is aripiprazole long-acting injection (LAI) administered every 2 months, 6 weeks, or 1 month, which is the only antipsychotic D_2_ partial agonist with an LAI formulation. It is followed by paliperidone LAI administered every 6 months. This antipsychotic has the least frequent administration schedule. According to the AFI, the most favorable antipsychotics for functionality are generally second-generation LAI antipsychotics. The D_2_ partial agonist mechanism has a pharmacodynamic advantage. Based on this functionality index, psychiatrists could select the most suitable antipsychotic for each patient, with the ultimate goal of helping the patient achieve their maximum potential.

## 1 Introduction

Schizophrenia, a severe psychiatric condition, often manifests during young adulthood and can lead to significant and long-lasting deficits in patient functionality (Patel et al., 2014). These deficits may be accompanied by elevated levels of cardiovascular (Correll et al., 2022) and metabolic (Pillinger et al., 2020) comorbidities, sudden death (Scorza et al., 2021), and reduced life expectancy (Hjorthøj et al., 2017). Globally, schizophrenia ranks among the top 25 causes of disability (Boland et al., 2022; Switaj et al., 2012).

In many traditional communities, the stigma associated with schizophrenia may affect the family as a whole, and it could also restrict, for instance, marital opportunities for younger family members (Boland et al., 2022).

Nonadherence rates are very high in schizophrenia, and it is estimated that 40%–50% of patients become at least partially noncompliant with treatment within 1 or 2 years. Due to the high risk of relapse and other potential consequences (job loss, interference with school, family burden, suicidality, homelessness, and aggressive or violent behavior), treatment adherence has become a critical issue for schizophrenia treatment (Boland et al., 2022). The concept of recovery in schizophrenia involves controlling both positive and negative symptoms and achieving an acceptable level of social and occupational functionality. Inadequate functioning may stem from residual negative and cognitive symptoms, which many antipsychotics do not adequately address, as they are more effective in managing positive symptomatology (Correll, 2020). It is widely acknowledged that sustained antipsychotic treatment is the only approach proven to be effective for achieving remission, maintaining it, and preventing relapses (Ifteni et al., 2021).

The antipsychotics currently in use span a spectrum ranging from silent antagonists of dopamine D_2_ receptors to nearly full agonists of the same receptors When an antipsychotic binds to D_2_ receptors, it blocks the action of dopamine on these receptors. If it has no other effect on D_2_ receptors, it is called silent antagonist or simply antagonist. If it has a stimulating effect on D_2_ receptors, but less than dopamine, it is called partial agonist. An overly intense agonist effect may fail to treat psychosis, as it cannot adequately control positive symptomatology. This is why partial agonists and silent antagonists of D_2_ receptors are preferable solutions. Blocking serotoninergic 5HT_2A_ receptors, which is achieved by many atypical antipsychotics, represents an important step in improving their tolerability. Additionally, certain antipsychotics may induce partial agonism in serotoninergic 5HT_1A_ receptors, providing additional benefits (Stahl, 2021).

The functionality and quality of life of patients with schizophrenia have been evaluated for various antipsychotics in clinical studies by comparing them to placebos and/or making direct comparisons (Ishigooka et al., 2022). Additional data have been obtained through meta-analyses and systematic reviews (Leucht et al., 2017; Huhn et al., 2019).

Antipsychotics differ in many parameters, such as pharmacodynamics, pharmacokinetics, posology, and pharmaceutical form, which provide both beneficial effects and inconveniences (Stahl and Djokic, 2023). These differences can influence the functionality of patients with schizophrenia (de Filippis et al., 2021).

The present study proposes a theoretical index that predicts the ability of an antipsychotic to improve the functionality of patients with schizophrenia. An advantage of this index is that it directly compares first- and second-generation antipsychotics.

## 2 Methodology

**FIGURE 1:**
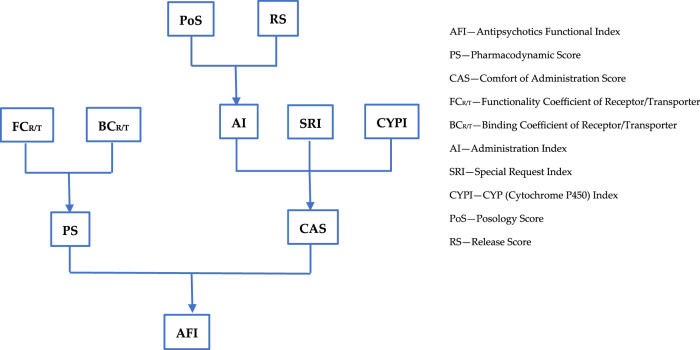
Antipsychotics Functional Index algorithm.

The impact of antipsychotics on patient functioning was analyzed, considering aspects such as pharmacodynamics, pharmacokinetics, pharmaceutical form, ease of administration, and the safety profile. A total of 29 antipsychotics used over time in the treatment of patients with schizophrenia were analyzed: chlorpromazine, flupenthixol, fluphenazine, haloperidol, loxapine, methotrimeprazine, periciazine, perphenazine, pimozide, thioridazine, thiothixene, trifluoperazine, zuclopenthixol, asenapine, clozapine, iloperidone, sertindole, lumateperone, lurasidone, olanzapine, zotepine, paliperidone, quetiapine, amisulpride, risperidone, ziprasidone, aripiprazole, brexpiprazole, and cariprazine. All these antipsychotics were compared to a hypothetical ideal antipsychotic characterized exclusively by beneficial effects known as the theoretical maximum. A parameter called the Antipsychotics Functional Index (AFI) was created according to the algorithm presented in Figure 1.

The general formula for the Antipsychotics Functional Index (AFI) is

$$AFI=\frac{PS+CAS}{2}=\frac{\sum \left({FC}_{R_{i}/T_{i}}\times {BC}_{R_{i}/T_{i}}\right)+AI+SRI+CYPI}{2}=\frac{\sum \left({FC}_{R_{i}/T_{i}}\times {BC}_{R_{i}/T_{i}}\right)+\frac{PoS+RS}{2}+SRI+CYPI}{2}$$

and all parameters included in it are defined and described below.

The Antipsychotics Functional Index (AFI) considers two major aspects, namely, pharmacodynamics and comfort of administration. These two aspects are each expressed using a score (percentage): the Pharmacodynamic Score (PS) and the Comfort of Administration Score (CAS).

### 2.1 Pharmacodynamic score (PS)

To assess effects on patient functionality from a pharmacodynamic perspective, the Pharmacodynamic Score (PS) was defined. This parameter takes into account the pharmacodynamic mechanisms of antipsychotics, which are split based on their pro-functioning and anti-functioning effects. To quantify these effects, a functionality coefficient (FC_R/T_) is calculated for each receptor/transporter. However, pharmacodynamic mechanisms are not equally addressed by antipsychotics. Thus, the binding coefficient (BC_R/T_) was created to quantify the degree to which the mechanisms are addressed. Each antipsychotic acts to varying degrees on multiple pharmacodynamic mechanisms. For this reason, the PS calculated for each antipsychotic represents a sum of scores corresponding to mechanisms that are involved to varying degrees. The receptor/transporter-specific score is calculated as the product of the corresponding FC_R/T_ and BC_R/T_. Consequently, the PS is calculated based on the following formula representing the sum of the products of the mentioned coefficients:

$$PS=\sum \left({FC}_{R/T}\times {BC}_{R/T}\right)$$

The functionality coefficient (FC_R/T_) represents the score associated with each receptor or reuptake pump (receptor/transporter) regarding functionality according to the following formula:

$${FC}_{R/T}={ProF\;Score\;}_{R/T}+{AntiF\;Score}_{R/T}$$

where F stands for functionality.

When calculating the FC_R/T_, both beneficial (rated positively) and unfavorable (rated negatively) actions regarding functionality are considered. Thus, for beneficial actions, scoring is carried out based on the importance of each effect, with values ranging from 3 to 1 according to Table 1. Actions of very high importance for patient functionality (improving positive symptoms, negative symptoms, and aggressiveness) each receive scores of 3. These actions are considered the most important as they alleviate the core symptoms of schizophrenia, and in their absence, functionality remains an unattainable goal. Pro-cognitive actions; improving sleep and motivation; and antidepressant, anxiolytic, and antimanic actions each receive scores of 2, as they are also important in the overall picture of functionality but are not parts of the core symptoms targeted by antipsychotics. Additional actions (improving some side effects and any other additional pro-functional effects) each receive scores of 1.

**TABLE 1:** The scores of favorable pharmacodynamic actions for functionality.

Pro-functionality action on receptor/transporter	Score
Reducing positive symptoms	3
Reducing negative symptoms	3
Anti-aggressiveness effect	3
Pro-cognitive effect	2
Sleep improvement	2
Motivation improvement	2
Antidepressant effect	2
Antimanic effect	2
Anxiolytic effect	2
Improving any adverse effect of an antipsychotic	1 (for each)
Any other pro-functionality effects	1 (for each)

Unfavorable actions affecting functionality are rated negatively, with their values determined based on the intensity of the adverse effects resulting from actions on receptors and reuptake pumps. The severities of adverse effects are rated based on the Merck Reporting Model (Severity-of-adverse-drug-reactions, 2024). This model is presented in Annex 2.

The adverse event scores, classified based on their severities, are presented in Table 2.

**TABLE 2:** Scores related to adverse events according to severity level.

Adverse event severity	Score
Mild	−0.25
Moderate	−0.5
Severe	−0.75
Lethal	−1

For each pharmacological mechanism, the total score of the unfavorable actions results from summing the products of the scores of each type of adverse effect and their numbers, yielding a negative value.

The pro- and anti-functionality actions and their corresponding scores, as well as the functionality coefficient specific to each action on receptors/reuptake pumps, can be found in Table 3.

**TABLE 3:** Functionality coefficients for receptors and transporters; NA—not applicable (Stahl, 2021; Procyshyn et al., 2023; Procyshyn et al., 2019).

Receptor/neurotransmitter pump action	Pro-functionality effects	Pro-functionality score	Anti-functionality effects	Anti-functionality score	Functionality coefficient of receptor/transporter (FC_R/T_)
Antagonism of postsynaptic D2 receptors	In mesolimbic/mesostriatal tract, reduction in positive symptoms, antimanic effect	5	– In nigrostriatal tract, EPS (acute dystonia, pseudoparkinsonism, akathisia, and tardive dyskinesia) and neuroleptic malignant syndrome – In tuberoinfundibular tract, prolactin elevation leading to galactorrhea, sexual dysfunction, infertility (especially in woman), demineralization of bones, and weight gain – In mesocortical/mesostriatal tract, may exacerbate negative symptoms, affective symptoms, and cognitive symptoms	−6.25	−1.25
Partial agonism of postsynaptic D2 receptors	Reduction in positive symptoms, improvement in negative symptoms, and reduction in hyperprolactinemia	7	Some akathisia	−0.5	6.5
Partial agonism/antagonism of D3 receptors	Antidepressant effect, improvement in negative symptoms, pro-cognitive effect, and motivation improvement	9	NA	0	9
Antagonism of H1 receptors	Anti-emetic effect and anxiolytic effects	3	Sedation, drowsiness, increase in appetite, weight gain, and postural hypotension	−1.75	1.25
Antagonism of M1 receptors	Mitigation of extrapyramidal adverse effects	1	Dry mouth, dry eyes, blurred vision, constipation, urinary retention, sinus tachycardia, QRS changes, confusion, worsening cognition, delirium, sedation, and exacerbation/attack of narrow-angle glaucoma Potentiation of effects of drugs with anticholinergic properties	−6	−5
Antagonism of M3 receptors	NA	0	Beta-cell failure, reduced insulin release, glucose intolerance, and type 2 diabetes mellitus	−2	−2
Antagonism of ⍺1 receptors	NA	0	Postural hypotension, dizziness, reflex tachycardia, and sedation	1.5	−1.5
Antagonism of ⍺2 receptors	– May improve cognitive deficits and have antidepressant effect – Antagonism of presynaptic α2-adrenergic receptors enhances serotonergic and noradrenergic transmission	4	Sexual dysfunction and priapism	−1.25	2.75
Antagonism/partial agonism of 5-HT1A receptors	Pro-cognitive, anxiolytic, antidepressant, and anti-aggressive effects	9	NA	0	9
Antagonism of 5-HT1B receptors	Antidepressant and pro-cognitive effects	4	NA	0	4
Antagonism of 5-HT1D receptors	Antidepressant effect	2	NA	0	2
Antagonism of 5-HT2A receptors	Ameliorates EPS; improves negative, cognitive, and mood-related symptoms; ameliorates hyperprolactinemia; improves positive symptoms; has anxiolytic and antimigraine effects; and improves sleep	16	Sedation, hypotension, and ejaculation problems	−1	15
Antagonism of 5-HT2C receptors	Pro-cognitive, antidepressant, and anxiolytic effects	6	Increased appetite and weight gain	−0.75	5.25
Antagonism of 5-HT3 receptors	Antidepressant and pro-cognitive effects, improves nausea and vomiting	6	NA	0	6
Antagonism of 5-HT6 receptors	antidepressant effect	2	NA	0	2
Antagonism of 5-HT7 receptors	Pro-cognitive, anxiolytic, and antidepressant effects	6	NA	0	6
Norepinephrine transporter (NET) inhibition	Antidepressant actions	2	Tremors, tachycardia, hypertension, sweating, insomnia, and erectile and ejaculation problems	−2.5	−0.5
Serotonin transporter (SERT) inhibition	Antidepressant actions and anti-anxiety, anti-panic, and anti-obsessional effects	4	Dyspepsia, nausea, headache, nervousness, akathisia, extrapyramidal effects, anorexia, and sexual side effects	−3	1

The binding coefficient (BC_R/T_) is a parameter that expresses the affinity of an antipsychotic for a receptor (R) and/or transporter (T). This parameter is necessary because antipsychotics have different affinities for different substrates, leading to effects with various amplitudes.

The binding coefficient ( ${BC}_{R/T}$ ) is directly proportional to the affinity of each antipsychotic for receptors and transporters. Thus, this parameter depends on the inhibition constant (*k*_*i*_), as they are inversely proportional (Table 4).

**TABLE 4:** The ${\text{BC}}_{R/T}$ values based on $k_{i}$ .

Binding coefficient of receptor/transporter (BC_R/T_)	K_i_ (nM)
1	0.001–1
0.8	1–10
0.6	10–100
0.4	100–1000
0.2	1000–10.000
0	>10.000

The values of *k*_*i*_ identified for the main antipsychotics (Procyshyn et al., 2023; PDSP, 2019) form the basis of the BC_R/T_ calculations (Annex 1). The theoretical maximum has a BC_R/T_ equal to 1 for receptors and transporters with positive values of FC_R/T_ (pro-functionality) and a BC_R/T_ equal to 0 for receptors and transporters with negative values of FC_R/T_ (anti-functionality). In the case of D_2_ receptors, the theoretical maximum is considered to be a partial agonist at this level (pro-functionality) rather than a silent antagonist.

The theoretical maximum PS is calculated for an ideal antipsychotic with a maximum effect for pharmacodynamic pro-functionality actions and an absence of any negative effect regarding functionality. The PS values corresponding to each antipsychotic are expressed as percentages of the theoretical maximum. The numerical PS values for the considered antipsychotics and the theoretical maximum, as well as the percentage values related to the theoretical maximum, are presented in Table 5.

**TABLE 5:** The pharmacodynamic score (PS and PS%).

Antipsychotic	PS	PS%
Chlorpromazine	25.6	42.14%
Flupenthixol	8.7	14.32%
Fluphenazine	32.05	52.76%
Haloperidol	20.7	34.07%
Loxapine	25.35	41.73%
Methotrimeprazine	12.5	20.58%
Periciazine	−0.45	−0.74%
Perphenazine	30.05	49.47%
Pimozide	28.3	46.58%
Thioridazine	26.25	43.21%
Thiothixene	27.65	45.51%
Trifluoperazine	19.05	31.36%
Zuclopenthixol	9.4	15.47%
Asenapine	45.85	75.47%
Clozapine	21.95	36.13%
Iloperidone	31	51.03%
Sertindole	34.6	56.95%
Lumateperone	17.1	28.15%
Lurasidone	34.7	57.12%
Olanzapine	24.25	39.92%
Zotepine	32.5	53.50%
Paliperidone	35.55	58.52%
Quetiapine	19.45	32.02%
Amisulpride	14.55	23.95%
Risperidone	35.8	58.93%
Ziprasidone	36.5	60.08%
Aripiprazole^(a)^	44.35	73.00%
Brexpiprazole^(a)^	46.6	76.71%
Cariprazine^(a)^	38.75	63.79%
Theoretical maximum	60.75	100.00%

### 2.2 Comfort of administration score (CAS)

The Comfort of Administration Score (CAS) quantifies how functionality can be influenced by the administration of an antipsychotic. The frequency of administration, the release of the active substance, any special requirements (e.g., post-administration surveillance, special investigations), and the influence of hepatic metabolic activity are taken into account. As for the Pharmacodynamic Score (PS), the Comfort of Administration Score (CAS) is calculated as a percentage of the theoretical maximum. The theoretical maximum is a score corresponding to an ideal antipsychotic that does not create any discomfort related to its administration (minimum administration frequency, constant release of the active substance, no special requirements related to administration, and no influence of liver metabolic activity).

The CAS considers the following criteria:

• The Administration Index (AI), which considers the frequency of administration and the release mode of the active substance.
• The Special Request Index (SRI), which quantifies the special requirements related to the administration of the antipsychotic.
• The CYP Index (CYPI), which expresses the potential for drug interactions generated at the level of cytochrome P450 (CYP450) enzymes.

The CAS represents the sum of these parameters according to the following formula:

CAS=AI+SRI+CYPI

The CAS formula equally considers the Administration Index (AI), the Special Request Index (SRI), and the CYP Index (CYPI), which will be discussed below.

The Administration Index (AI) is a parameter that evaluates how the patient’s comfort is influenced by the dosage and pharmaceutical form of the antipsychotic. To obtain this index, two other parameters are defined, namely, the Posology Score (PoS) and Release Score (RS). The formula for the AI is

$$AI=\frac{PoS+RS}{2}$$

Meaning the arithmetic mean of the PoS and RS, as they are thought to influence the AI with equal weights.

• The Posology Score (PoS) quantifies how functionality is influenced by the frequency of administration of the antipsychotic. It is thought that infrequent administrations are more protective regarding functionality (e.g., long-acting injectable antipsychotics), while more frequent administrations generate a negative influence. The formula for this parameter is

$$PoS=\frac{365,25-NAY}{365,25}$$

where NAY represents the Number of Administrations per Year and the value 365.25 results from the average number of days in a year, considering leap years.

The Number of Administrations per Year (NAY) is calculated taking into account administrations that occur once or multiple times a day (oral antipsychotics) or after a specific number of weeks, monthly, or every few months (LAI antipsychotics).

For example, for an antipsychotic administered twice daily (BID), the formula becomes

$$PoS=\frac{365,25-2\times 365,25}{365,25}=-1$$

Long-acting injection (LAI) antipsychotics have the highest values for this parameter (close to 1). The resulting values for each antipsychotic are found in Annex 4. It is important to mention that, in this analysis, it is assumed that a patient’s loss of personal comfort is proportional to the number of administrations. The ideal antipsychotic with which the final comparison is made is one that does not create any discomfort related to administration, which theoretically means zero administrations per year and a theoretical PoS = 1. This is currently impossible but necessary as a reference level for the analysis at hand.

• The Release Score (RS) quantifies the patient’s comfort regarding the medication’s release form. It is based on the premise that orally administered immediate-release substances can generate adverse effects due to greater fluctuations in plasma concentrations, unlike orally administered modified-release forms (extended release) and injectable forms with prolonged release (LAI), for which the smallest plasma fluctuations have been observed. An example would be quetiapine, which is available in both immediate-release and extended-release forms; it is much better tolerated and easier to titrate in the extended-release form. In our computations, the RS has the following values: immediate release = 0, extended release = 0.5, and LAI = 1. An ideal antipsychotic automatically receives a theoretical maximum score of 1 (Annex 4).

The Special Request Index (SRI) quantifies the presence or absence of special administration requirements (e.g., post-administration surveillance for 3 h after olanzapine LAI, ECG monitoring for sertindole, monitoring the number of platelets for clozapine), which in turn influence the patient’s comfort. Thus, the presence of special administration requirements results in a score of 0, while their absence results in a score of 1. An ideal antipsychotic also receives a theoretical maximum score of 1 (Annex 4).

The CYP Index (CYPI) analyzes how the patient’s functionality is influenced by the effect of an antipsychotic on the activity of cytochrome P450 enzymes (CYP). If an antipsychotic affects the activity of cytochrome enzymes by inhibiting or inducing them, this represents an obstacle or requires a dosage adjustment for other concomitant medications metabolized by the same enzymatic system.

Therefore, influencing cytochrome enzymes, regardless of the direction in which it occurs, results in a score of 0, while the absence of influence receives a score of 1. The ideal antipsychotic does not influence the cytochrome enzymatic system in any way, and it receives the theoretical maximum score of 1 (Annex 4).

The CAS values corresponding to the considered antipsychotics are presented in Annex 4.

Taking all these variables into account, we reach the final formula:

$$AFI\;\left(\%\right)=\frac{PS\;\left(\%\right)+CAS\;\left(\%\right)}{2}$$

**FIGURE 2:**
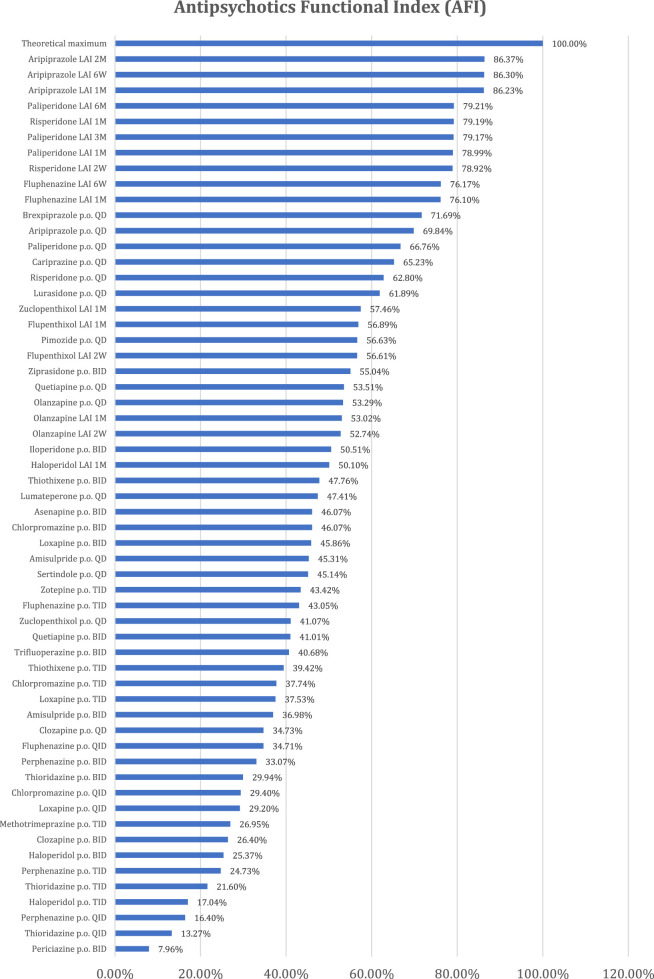
The Antipsychotics Functional Index (AFI) values for each antipsychotic and pharmaceutical form.

The resulting values for the Antipsychotics Functional Index (AFI) are presented in Annex 3 and Figure 2.

## 3 Discussion

The Pharmacodynamic Score (PS), which evaluates the impact of pharmacodynamic aspects on patient functionality, presents greater values for brexpiprazole, asenapine, aripiprazole, and cariprazine. It was expected that partial agonists of D_2_ receptors would be the most favorable for functionality due to their particular mode of action, which counterbalances the specific dopaminergic imbalances of the disease, thus offering efficacy and tolerability, essential aspects of functionality. The classification of asenapine among partial D_2_ agonists is justified by its favorable receptor profile. It has the maximum affinity for 5-HT_2A_ (the highest-rated pro-functionality receptor), 5-HT_2C_, 5-HT_6_, and 5-HT_7_ receptors, as well as very good affinity, almost at the maximum, for D_3_, 5-HT_1A_, and 5-HT_1B_ receptors, which are also favorable for patient functionality. Second-generation antipsychotics are generally better positioned compared to those of the first generation, with some exceptions such as amisulpride, quetiapine, clozapine, and olanzapine. The latter, although proven effective in schizophrenia, are not the most favorable for functionality due to less favorable pro- and anti-functionality ratios.

From the perspective of administration comfort (CAS), the best-ranked antipsychotics are those that achieve optimal administration with minimal frequency, have release modes that do not lead to large fluctuations in plasma concentrations, do not have special administration requirements, and lack interactions with cytochrome enzyme systems. Thus, the best-ranked antipsychotics are long-acting injectables (LAIs) (paliperidone every 6 months, paliperidone every 3 months, aripiprazole every 2 months, and aripiprazole every 6 weeks). Antipsychotics with unfavorable CASs require oral administration multiple times a day, have immediate-release forms, have special administration requirements, and have significant influence on the cytochrome enzyme system. In the cases of thioridazine (p.o. QID) and perphenazine (p.o. QID), negative values are recorded because they meet three of the four conditions mentioned earlier, namely, frequent daily administrations, immediate release, and inhibition of the CYP2D6 enzyme.

The Antipsychotics Functional Index (AFI) is higher for antipsychotics with both high Pharmacodynamic Scores (PSs), which are only correlated with the active substance, and good Comfort of Administration Scores (CASs), which are correlated with both the pharmaceutical forms of antipsychotics and the active substance. Consequently, the best ranking is obtained by the only antipsychotic dopaminergic D_2_ partial agonist with an LAI formulation (aripiprazole LAI), followed by the atypical LAI antipsychotic with the rarest administrations (paliperidone LAI administered every 6 M). Given this ranking, a better antipsychotic would result from combining a partial dopaminergic agonist with the lowest possible administration frequency.

Although risperidone and paliperidone are pharmacodynamically close, the profile of risperidone is more favorable to functionality. Therefore, risperidone with monthly administration is better positioned than paliperidone with administration every 3 months or 1 month.

Flupenthixol LAI ranks among the atypical antipsychotics in the final AFI ranking, combining a favorable PS (the best among first-generation antipsychotics) with administration every 6 weeks or 1 month, no special administration requirements, and no influence on cytochrome enzyme systems. In its oral-administration variant, flupenthixol no longer maintains the same favorable position for functionality.

Strictly analyzing oral antipsychotics, brexpiprazole is the most favorable for functionality, with the best PS, followed by aripiprazole, paliperidone, cariprazine, risperidone, and lurasidone, which all rank well.

Interestingly, oral olanzapine (QD) is better positioned compared to olanzapine LAI administered monthly or every 2 weeks. This is explained by the special requirements for olanzapine LAI administration (monitoring for 3 h after administration) (Medscape, 2024; Union Register of medicinal products, 2024).

An important influence on functionality is the cognitive and negative symptoms severity. Typical antipsychotics (e.g., haloperidol) decrease dopaminergic activity in the mesocortical tract, that ends in the prefrontal cortex, through D_2_ receptor antagonism, leading not only to the lack of improvement of these symptoms, but also to a possible worsening. An important step in the beneficial approach to negative and cognitive symptoms was the development of atypical antipsychotics (e.g., risperidone, olanzapine, clozapine) which modulate serotoninergic activity (5-HT_2A_ receptor antagonism with or without 5-HT_1A_ receptor partial agonism) and thus compensating for their tendency to decrease dopaminergic activity in the prefrontal cortex. A more direct way to improve the low prefrontal dopaminergic activity in schizophrenia is through D_2_ partial agonists (e.g., aripiprazole, cariprazine, brexpiprazole). They bind to free and unstimulated D_2_ receptors in the prefrontal cortex schizophrenia patients, leading through their action as D_2_ partial agonists to increase dopamine activity in this area and finally to the improvement of cognitive and negative symptoms. D_2_ partial agonists also retain the activity of modulating serotonergic receptors, thus improving cognitive and negative symptoms through three mechanisms: D _2_ partial agonism, 5-HT_2A_ antagonism and 5-HT_1A_ partial agonism. Based on these actions, they may be theoretically superior to atypical antipsychotics that combine D_2_ antagonism with serotonergic receptor modulation (Stahl, 2021).

The challenge of this theoretical, predictive concept lies in referencing existing data in the specialized literature, including meta-analyses and systematic reviews, which scrutinize reported clinical studies. It is crucial to confront predictions with observations. Consequently, we note that in a meta-analysis by Leucht et al. (2017), in terms of quality of life, aripiprazole, quetiapine, lurasidone, cariprazine, olanzapine, and paliperidone were identified as the most effective, while in terms of social functioning, thioridazine, lurasidone, olanzapine, risperidone, paliperidone, brexpiprazole, and aripiprazole ranked the best. In a meta-analysis conducted by Huhn et al. (2019), aripiprazole and paliperidone topped the rankings in terms of quality of life, whereas in terms of social functioning, thioridazine, olanzapine, paliperidone, quetiapine, lurasidone, and brexpiprazole were the most effective antipsychotics.

The STAR study, which compared oral antipsychotics, found that switching to oral aripiprazole from other oral antipsychotics led to improvements in negative symptoms, somnolence, weight gain, cognitive function, vitality, and mood (Kerwin et al., 2007).

Naber et al. (2015), Naber et al. (2018) provided additional data regarding the functional benefits following treatment with LAI antipsychotics, namely, aripiprazole LAI (1M) and paliperidone LAI (1M). In those studies, aripiprazole LAI demonstrated greater favorability in improving functional outcomes in patients with schizophrenia, particularly in those under 35 years old. Moreover, compared cariprazine vs. risperidone in terms of negative symptoms assessed throughout Positive and Negative Syndrome Scale - Factor Score for Negative Symptoms (PANSS-FSNS) and functionality assessed throughout Personal and Social Performance Scale (PSP), with a clear, statistic significance, superiority for cariprazine (Németh et al., 2017).

Ifteni et al. (2021) proposed the ROLIN scale with the aim of identifying patients who would benefit the most from LAI treatment. This tool considers a range of predictors of good or poor therapeutic outcomes, including age, duration of illness, number of relapses, the therapeutic response to oral antipsychotics, social support for the patient, the pharmaceutical form of the antipsychotic, and therapeutic adherence. Some of these predictors overlap with those considered when calculating the AFI. Thus, the combined use of the ROLIN scale and the AFI by a psychiatrist could provide additional benefits in the treatment of patients with schizophrenia.

Although the theoretical predictive index presented in this paper cannot perfectly align with the data resulting from these studies and meta-analyses, we cannot overlook the fact that in the resulting classification, aripiprazole, paliperidone, risperidone, brexpiprazole, cariprazine, and lurasidone are ranked at the top. An advantage of this predictive index could be that it also considers the pharmaceutical form in which the antipsychotic molecule is presented. Thus, two different methods, including a theoretical method that directly compares antipsychotics and another based on important clinical data, lead to comparable results. Nevertheless, theoretical data which result from our analysis should be correlated with clinical one through clinical functionality scales. Such scales as Heinrichs-Carpenter Quality of Life Scale, Global Assessment of Functionality Scale and others, will provide the real status of patient’s functionality and could be used to compare antipsychotics in terms of functionality.

## 4 Limitations

The classification of antipsychotics based on AFI represents a personal view of authors. The scores given have sought to take into account all aspects, from efficacy to safety profile, even the pharmaceutical form. However, other visions are possible, with scores conceived in a different way that could change this classification.

In the development of the AFI, only adverse reactions determined by pharmacodynamic action were considered. Adverse reactions based on reports and those mentioned in the summary of product characteristics (SPCs) of each antipsychotic were not taken into consideration. Although a rigorous analysis of the adverse reactions mentioned in the SPCs was conducted, it could not be used because it led to paradoxical results, namely, a superior safety profile for first-generation (typical) antipsychotics compared to second-generation (atypical) antipsychotics. This result was obtained because adverse reactions were reported much more rigorously during the development of atypical antipsychotics and thereafter in their marketing, in accordance with stricter pharmacovigilance legislation. Therefore, in this work, their inclusion in the final formula was abandoned, and the index remained reliant on theoretical and predictive parameters.

## 5 Conclusion

According to the Antipsychotics Functional Index (AFI), atypical LAI antipsychotics are the most favorable for the functionality of patients with schizophrenia. Among them, the partial agonist mechanism of D_2_ dopamine is advantageous.

Based on this functionality index, a clinical psychiatrist could, either from the beginning of treatment or during treatment, select the most suitable antipsychotic for their patient, with the ultimate goal of helping the patient achieve their maximum potential.

## Data Availability

The original contributions presented in the study are included in the article/Supplementary Material, further inquiries can be directed to the corresponding author/s.
