# Correction: The antipsychotics functional index (AFI) in schizophrenia

**DOI:** 10.3389/fphar.2025.1662228

**Published:** 2025-07-18

**Authors:** Gabriel-Cristian Marinescu, Petru Iulian Ifteni, Andreea Teodorescu, Radu Georgescu

**Affiliations:** ^1^ Dr. Marinescu G Gabriel-Cristian CMI, Pitești, Romania; ^2^ Faculty of Medicine, Transilvania University of Braşov, Braşov, Romania; ^3^ Department of Psychiatry, University of Medicine and Pharmacy “Iuliu Hațieganu” Cluj-Napoca, Cluj-Napoca, Romania

**Keywords:** schizophrenia, antipsychotics, functionality, AFI, LAI

**Affiliations** 1, 2 and 3 were erroneously given as 1. Dr. Marinescu G Gabriel-Cristian CMI, Pitesti, Romania; 2. Clinical Hospital of Psychiatry and Neurology Braşov, Transilvania University of Braşov, Brasov, Romania; 3. Department of Psychiatry, University of Medicine and Pharmacy “Iuliu Haieganu” Cluj-Napoca, Cluj-Napoca, Romania.

The original version of this article has been updated.

