# Supplementary material for: The antipsychotics functional index (AFI) in schizophrenia
Source: Front Pharmacol. 2025 Jul 2;16:1591763. doi: 10.3389/fphar.2025.1591763 (PMC12264984; doi:10.3389/fphar.2025.1591763)
Supplement: Supplementary file 1 [file Supplementaryfile1.docx]

**Annex 1 –** $\boldsymbol{FC}_{\boldsymbol{R/T}}$ **și** $\boldsymbol{BC}_{\boldsymbol{R/T}}$ **values of antipsychotics with the theoretically maximum**

|  | **D_2_ blockade/partial agonist** | | **D_3_ blockade/partial agonist** | | **H_1_ blockade** | | **M_1_ blockade** | | **M_3_ blockade** | | **α_1_ blockade** | | **α_2_ blockade** | | **5-HT_1A_ blockade** | | **5-HT_1B_ blockade** | | **5-HT_2A_ blockade** | | **5-HT_2C_ blockade** | | **5-HT_6_ blockade** | | **5-HT_7_ blockade** | |
| --- | --- | --- | --- | --- | --- | --- | --- | --- | --- | --- | --- | --- | --- | --- | --- | --- | --- | --- | --- | --- | --- | --- | --- | --- | --- | --- |
|  | FC_R/T_ | BC_R/T_ | FC_R/T_ | BC_R/T_ | FC_R/T_ | BC_R/T_ | FC_R/T_ | BC_R/T_ | FC_R/T_ | BC_R/T_ | FC_R/T_ | BC_R/T_ | FC_R/T_ | BC_R/T_ | FC_R/T_ | BC_R/T_ | FC_R/T_ | BC_R/T_ | FC_R/T_ | BC_R/T_ | FC_R/T_ | BC_R/T_ | FC_R/T_ | BC_R/T_ | FC_R/T_ | BC_R/T_ |
| Chlorpromazine | -1.25 | 0.8 | 9 | 0.8 | 1.25 | 0.6 | -5 | 0.6 | -2 | 0.6 | -1.5 | 0.8 | 2.75 | 0.4 | 9 | 0.2 | 4 | 0.2 | 15 | 0.8 | 5.25 | 0.6 | 2 | 0.8 | 6 | 0.6 |
| Flupenthixol | -1.25 | 1 | 9 | 0 | 1.25 | 0.6 | -5 | 0.6 | -2 | 0 | -1.5 | 0.6 | 2.75 | 0.4 | 9 | 0 | 4 | 0 | 15 | 0.8 | 5.25 | 0 | 2 | 0 | 6 | 0 |
| Fluphenazine | -1.25 | 1 | 9 | 1 | 1.25 | 0.6 | -5 | 0.2 | -2 | 0.2 | -1.5 | 0.6 | 2.75 | 0.2 | 9 | 0.4 | 4 | 0.4 | 15 | 0.8 | 5.25 | 0.4 | 2 | 0.6 | 6 | 0.8 |
| Haloperidol | -1.25 | 1 | 9 | 0.8 | 1.25 | 0.2 | -5 | 0.2 | -2 | 0.2 | -1.5 | 0.6 | 2.75 | 0.2 | 9 | 0.2 | 4 | 0.4 | 15 | 0.6 | 5.25 | 0.2 | 2 | 0.2 | 6 | 0.4 |
| Loxapine | -1.25 | 0.8 | 9 | 0.6 | 1.25 | 0.6 | -5 | 0.4 | -2 | 0.4 | -1.5 | 0.6 | 2.75 | 0.2 | 9 | 0.2 | 4 | 0.4 | 15 | 0.8 | 5.25 | 0.6 | 2 | 0.6 | 6 | 0.6 |
| Methotrimeprazine | -1.25 | 0.6 | 9 | 0 | 1.25 | 1 | -5 | 0 | -2 | 0 | -1.5 | 0 | 2.75 | 0 | 9 | 0 | 4 | 0 | 15 | 0.8 | 5.25 | 0 | 2 | 0 | 6 | 0 |
| Periciazine | -1.25 | 0.8 | 9 | 0 | 1.25 | 0 | -5 | 0 | -2 | 0 | -1.5 | 0 | 2.75 | 0.2 | 9 | 0 | 4 | 0 | 15 | 0 | 5.25 | 0 | 2 | 0 | 6 | 0 |
| Perphenazine | -1.25 | 1 | 9 | 1 | 1.25 | 0.8 | -5 | 0.2 | -2 | 0.2 | -1.5 | 0.6 | 2.75 | 0.4 | 9 | 0.4 | 4 | 0 | 15 | 0.8 | 5.25 | 0.4 | 2 | 0.6 | 6 | 0.6 |
| Pimozide | -1.25 | 0.8 | 9 | 1 | 1.25 | 0.2 | -5 | 0.2 | -2 | 0 | -1.5 | 0.6 | 2.75 | 0.4 | 9 | 0.4 | 4 | 0 | 15 | 0.6 | 5.25 | 0.2 | 2 | 0.6 | 6 | 1 |
| Thioridazine | -1.25 | 0.8 | 9 | 0.8 | 1.25 | 0.6 | -5 | 0.8 | -2 | 0.6 | -1.5 | 0.8 | 2.75 | 0.2 | 9 | 0.4 | 4 | 0.4 | 15 | 0.8 | 5.25 | 0.6 | 2 | 0.6 | 6 | 0.6 |
| Thiothixene | -1.25 | 1 | 9 | 1 | 1.25 | 0.6 | -5 | 0.2 | -2 | 0 | -1.5 | 0.4 | 2.75 | 0.4 | 9 | 0.4 | 4 | 0.4 | 15 | 0.6 | 5.25 | 0.2 | 2 | 0.4 | 6 | 0.6 |
| Trifluoperazine | -1.25 | 0.8 | 9 | 0 | 1.25 | 0.4 | -5 | 0.2 | -2 | 0 | -1.5 | 0.6 | 2.75 | 0.2 | 9 | 0.4 | 4 | 0 | 15 | 0.8 | 5.25 | 0.4 | 2 | 0.4 | 6 | 0.4 |
| Zuclopenthixol | -1.25 | 1 | 9 | 0 | 1.25 | 0.6 | -5 | 0.4 | -2 | 0 | -1.5 | 0.8 | 2.75 | 0.4 | 9 | 0 | 4 | 0 | 15 | 0.8 | 5.25 | 0 | 2 | 0 | 6 | 0 |
| Asenapine | -1.25 | 0.8 | 9 | 0.8 | 1.25 | 0.8 | -5 | 0.2 | -2 | 0 | -1.5 | 0.8 | 2.75 | 0.8 | 9 | 0.8 | 4 | 0.8 | 15 | 1 | 5.25 | 1 | 2 | 1 | 6 | 1 |
| Clozapine | -1.25 | 0.4 | 9 | 0.4 | 1.25 | 0.8 | -5 | 0.6 | -2 | 0.6 | -1.5 | 0.8 | 2.75 | 0.4 | 9 | 0.4 | 4 | 0.4 | 15 | 0.6 | 5.25 | 0.6 | 2 | 0.6 | 6 | 0.6 |
| Iloperidone | -1.25 | 0.6 | 9 | 0.6 | 1.25 | 0.4 | -5 | 0.2 | -2 | 0.2 | -1.5 | 0.8 | 2.75 | 0.4 | 9 | 0.4 | 4 | 0.6 | 15 | 1 | 5.25 | 0.6 | 2 | 0.4 | 6 | 0.4 |
| Sertindole | -1.25 | 0.8 | 9 | 0.8 | 1.25 | 0.4 | -5 | 0.4 | -2 | 0.2 | -1.5 | 0.8 | 2.75 | 0.4 | 9 | 0.4 | 4 | 0.6 | 15 | 1 | 5.25 | 0.8 | 2 | 0.8 | 6 | 0.6 |
| Lumateperone | -1.25 | 0.6 | 9 | 0 | 1.25 | 0 | -5 | 0 | -2 | 0 | -1.5 | 0.6 | 2.75 | 0.6 | 9 | 0 | 4 | 0 | 15 | 1 | 5.25 | 0.4 | 2 | 0 | 6 | 0 |
| Lurasidone | -1.25 | 0.8 | 9 | 0.6 | 1.25 | 0.2 | -5 | 0.2 | -2 | 0 | -1.5 | 0.6 | 2.75 | 0.6 | 9 | 0.8 | 4 | 0 | 15 | 1 | 5.25 | 0.4 | 2 | 0 | 6 | 1 |
| Olanzapine | -1.25 | 0.6 | 9 | 0.6 | 1.25 | 0.8 | -5 | 0.8 | -2 | 0.6 | -1.5 | 0.6 | 2.75 | 0.4 | 9 | 0.2 | 4 | 0.4 | 15 | 0.8 | 5.25 | 0.8 | 2 | 0.8 | 6 | 0.4 |
| Zotepine | -1.25 | 0.6 | 9 | 0.8 | 1.25 | 0.8 | -5 | 0.4 | -2 | 0.4 | -1.5 | 0.8 | 2.75 | 0.6 | 9 | 0.4 | 4 | 0.6 | 15 | 0.8 | 5.25 | 0.8 | 2 | 0.8 | 6 | 0.6 |
| Paliperidone | -1.25 | 0.8 | 9 | 0.8 | 1.25 | 0.6 | -5 | 0 | -2 | 0 | -1.5 | 0.8 | 2.75 | 0.6 | 9 | 0.4 | 4 | 0.6 | 15 | 1 | 5.25 | 0.6 | 2 | 0.2 | 6 | 0.6 |
| Quetiapine | -1.25 | 0.4 | 9 | 0.4 | 1.25 | 0.6 | -5 | 0.4 | -2 | 0.2 | -1.5 | 0.6 | 2.75 | 0.6 | 9 | 0.4 | 4 | 0.2 | 15 | 0.6 | 5.25 | 0.2 | 2 | 0.2 | 6 | 0.4 |
| Amisulpride | -1.25 | 0.8 | 9 | 0.8 | 1.25 | 0 | -5 | 0 | -2 | 0 | -1.5 | 0 | 2.75 | 0.2 | 9 | 0 | 4 | 0.2 | 15 | 0.2 | 5.25 | 0 | 2 | 0.2 | 6 | 0.6 |
| Risperidone | -1.25 | 0.8 | 9 | 0.8 | 1.25 | 0.6 | -5 | 0 | -2 | 0.2 | -1.5 | 0.8 | 2.75 | 0.4 | 9 | 0.4 | 4 | 0.6 | 15 | 1 | 5.25 | 0.6 | 2 | 0.2 | 6 | 0.8 |
| Ziprasidone | -1.25 | 0.8 | 9 | 0.8 | 1.25 | 0.6 | -5 | 0 | -2 | 0.2 | -1.5 | 0.6 | 2.75 | 0.4 | 9 | 0.6 | 4 | 0.8 | 15 | 0.8 | 5.25 | 0.6 | 2 | 0.6 | 6 | 0.8 |
| Aripiprazole^(a)^ | 6.5 | 1 | 9 | 0.8 | 1.25 | 0.6 | -5 | 0 | -2 | 0.2 | -1.5 | 0.6 | 2.75 | 0.6 | 9 | 0.8 | 4 | 0.4 | 15 | 0.8 | 5.25 | 0.6 | 2 | 0.4 | 6 | 0.8 |
| Brexpiprazole^(a)^ | 6.5 | 1 | 9 | 0.8 | 1.25 | 0.6 | -5 | 0.2 | -2 | 0 | -1.5 | 0.6 | 2.75 | 0.6 | 9 | 1 | 4 | 0.6 | 15 | 1 | 5.25 | 0 | 2 | 0.6 | 6 | 0.8 |
| Cariprazine^(a)^ | 6.5 | 1 | 9 | 1 | 1.25 | 0.6 | -5 | 0 | -2 | 0 | -1.5 | 0.4 | 2.75 | 0 | 9 | 0.8 | 4 | 0.4 | 15 | 0.6 | 5.25 | 0.4 | 2 | 0.4 | 6 | 0.4 |
| Theoretical maximum | 6.5 | 1 | 9 | 1 | 1.25 | 1 | -5 | 0 | -2 | 0 | -1.5 | 0 | 2.75 | 1 | 9 | 1 | 4 | 1 | 15 | 1 | 5.25 | 1 | 2 | 1 | 6 | 1 |

1. D_2_ partial agonist antipsychotic
