# Supplementary material for: The antipsychotics functional index (AFI) in schizophrenia
Source: Front Pharmacol. 2025 Jul 2;16:1591763. doi: 10.3389/fphar.2025.1591763 (PMC12264984; doi:10.3389/fphar.2025.1591763)
Supplement: Supplementary file 2 [file Supplementaryfile3.docx]

**Annex 3 – Antipsychotics Functional Index (AFI) values**

| **Antipsychotic** | **Regimen** | **PS (%)** | **CAS (%)** | **AFI (%)** |
| --- | --- | --- | --- | --- |
| Chlorpromazine | p.o. BID | 42,14 | 50,00 | 46,07 |
| Chlorpromazine | p.o. TID | 42,14 | 33,33 | 37,74 |
| Chlorpromazine | p.o. QID | 42,14 | 16,67 | 29,40 |
| Flupenthixol | LAI 2W | 14,32 | 98,90 | 56,61 |
| Flupenthixol | LAI 1M | 14,32 | 99,45 | 56,89 |
| Fluphenazine | p.o. QID | 52,76 | 16,67 | 34,71 |
| Fluphenazine | p.o. TID | 52,76 | 33,33 | 43,05 |
| Fluphenazine | LAI 1M | 52,76 | 99,45 | 76,10 |
| Fluphenazine | LAI 6W | 52,76 | 99,59 | 76,17 |
| Haloperidol | p.o. BID | 34,07 | 16,67 | 25,37 |
| Haloperidol | p.o. TID | 34,07 | 0,00 | 17,04 |
| Haloperidol | LAI 1M | 34,07 | 66,12 | 50,10 |
| Loxapine | p.o. BID | 41,73 | 50,00 | 45,86 |
| Loxapine | p.o. TID | 41,73 | 33,33 | 37,53 |
| Loxapine | p.o. QID | 41,73 | 16,67 | 29,20 |
| Methotrimeprazine | p.o. TID | 20,58 | 33,33 | 26,95 |
| Periciazine | p.o. BID | -0,74 | 16,67 | 7,96 |
| Perphenazine | p.o. QID | 49,47 | -16,67 | 16,40 |
| Perphenazine | p.o. TID | 49,47 | 0,00 | 24,73 |
| Perphenazine | p.o. BID | 49,47 | 16,67 | 33,07 |
| Pimozide | p.o. QD | 46,58 | 66,67 | 56,63 |
| Thioridazine | p.o. QID | 43,21 | -16,67 | 13,27 |
| Thioridazine | p.o. TID | 43,21 | 0,00 | 21,60 |
| Thioridazine | p.o. BID | 43,21 | 16,67 | 29,94 |
| Thiothixene | p.o. BID | 45,51 | 50,00 | 47,76 |
| Thiothixene | p.o. TID | 45,51 | 33,33 | 39,42 |
| Trifluoperazine | p.o. BID | 31,36 | 50,00 | 40,68 |
| Zuclopenthixol | p.o. QD | 15,47 | 66,67 | 41,07 |
| Zuclopenthixol | LAI 1M | 15,47 | 99,45 | 57,46 |
| Asenapine | p.o. BID | 75,47 | 16,67 | 46,07 |
| Clozapine | p.o. QD | 36,13 | 33,33 | 34,73 |
| Clozapine | p.o. BID | 36,13 | 16,67 | 26,40 |
| Iloperidone | p.o. BID | 51,03 | 50,00 | 50,51 |
| Sertindole | p.o. QD | 56,95 | 33,33 | 45,14 |
| Lumateperone | p.o. QD | 28,15 | 66,67 | 47,41 |
| Lurasidone | p.o. QD | 57,12 | 66,67 | 61,89 |
| Olanzapine | p.o. QD | 39,92 | 66,67 | 53,29 |
| Olanzapine | LAI 2W | 39,92 | 65,57 | 52,74 |
| Olanzapine | LAI 1M | 39,92 | 66,12 | 53,02 |
| Zotepine | p.o. TID | 53,50 | 33,33 | 43,42 |
| Paliperidone | p.o. QD | 58,52 | 75,00 | 66,76 |
| Paliperidone | LAI 1M | 58,52 | 99,45 | 78,99 |
| Paliperidone | LAI 3M | 58,52 | 99,82 | 79,17 |
| Paliperidone | LAI 6M | 58,52 | 99,91 | 79,21 |
| Quetiapine | p.o. BID | 32,02 | 50,00 | 41,01 |
| Quetiapine | p.o. QD | 32,02 | 75,00 | 53,51 |
| Amisulpride | p.o. QD | 23,95 | 66,67 | 45,31 |
| Amisulpride | p.o. BID | 23,95 | 50,00 | 36,98 |
| Risperidone | p.o. QD | 58,93 | 66,67 | 62,80 |
| Risperidone | LAI 2W | 58,93 | 98,90 | 78,92 |
| Risperidone | LAI 1M | 58,93 | 99,45 | 79,19 |
| Ziprasidone | p.o. BID | 60,08 | 50,00 | 55,04 |
| Aripiprazole | p.o. QD | 73,00 | 66,67 | 69,84 |
| Aripiprazole | LAI 1M | 73,00 | 99,45 | 86,23 |
| Aripiprazole | LAI 6W | 73,00 | 99,59 | 86,30 |
| Aripiprazole | LAI 2M | 73,00 | 99,73 | 86,37 |
| Brexpiprazole | p.o. QD | 76,71 | 66,67 | 71,69 |
| Cariprazine | p.o. QD | 63,79 | 66,67 | 65,23 |
| Theoretical maximum | ideal* | 100,00 | 100,00 | 100,00 |
