# Supplementary material for: The antipsychotics functional index (AFI) in schizophrenia
Source: Front Pharmacol. 2025 Jul 2;16:1591763. doi: 10.3389/fphar.2025.1591763 (PMC12264984; doi:10.3389/fphar.2025.1591763)
Supplement: Supplementary file 3 [file Supplementaryfile2.docx]

**Annex 2: MSD Manual Severity of Adverse Drug Reactions**

Adverse drug reactions (adverse effects) are any unwanted effects of drugs (including medications). There is no universal scale for describing or measuring the severity of an adverse drug reaction. Assessment is largely subjective. Reactions can be described as:

**Mild**

**Moderate**

**Severe**

**Lethal (deadly)**

**Mild or moderate** adverse drug reactions do not necessarily mean that people must stop taking a medication, especially if no suitable alternative is available. However, doctors are likely to reevaluate the dose, frequency of use (number of doses a day), and timing of doses (for example, before or after meals; in the morning or at bedtime). Other medications may be used to control the adverse drug reaction (for example, a stool softener to relieve constipation).

**Mild adverse drug reactions**

Mild reactions usually described as of minor significance include

Digestive disturbances (such as nausea, constipation, diarrhea)

Headaches

Fatigue

Vague muscle aches

Malaise (a general feeling of illness or discomfort)

Changes in sleep patterns

However, such reactions can be very distressing to people who experience them. As a result, people may be less willing to take their medication as instructed, and the goals of treatment may not be achieved.

**Moderate adverse drug reactions**

Moderate reactions include

Rashes (especially if they are extensive and persistent)

Visual disturbances (especially in people who wear corrective lenses)

Muscle tremor

Difficulty with urination (a common effect of many medications in older men)

Any perceptible change in mood or mental function

Certain changes in blood components, such as a temporary, reversible decrease in the white blood cell count or in blood levels of some substances, such as glucose

Also, reactions that are usually described as mild are considered moderate if the person experiencing them considers them distinctly annoying, distressing, or intolerable.

**Severe adverse drug reactions**

Severe reactions include those that may be life threatening (such as liver failure, abnormal heart rhythms, certain types of allergic reactions), those that result in persistent or significant disability or hospitalization, and those that cause birth defects. Severe reactions are relatively rare. People who develop a severe reaction usually must stop using the drug or medication and must be treated. However, doctors must sometimes continue giving high-risk medications (for example, chemotherapy to people with cancer or immunosuppressants to people undergoing organ transplantation). Doctors use every possible means to control a severe adverse drug reaction.

**Lethal adverse drug reactions**

Lethal reactions are those in which a drug reaction directly or indirectly caused death. These reactions are typically severe reactions that were not detected in time or did not respond to treatment. Lethal reactions can be the reasons that some medications are withdrawn from the market (such as troglitazone and terfenadine).
