# Supplementary material for: The antipsychotics functional index (AFI) in schizophrenia
Source: Front Pharmacol. 2025 Jul 2;16:1591763. doi: 10.3389/fphar.2025.1591763 (PMC12264984; doi:10.3389/fphar.2025.1591763)
Supplement: Supplementary file 4 [file Supplementaryfile4.docx]

**Annex 4: CAS values of antipsychotics and parameters included in its formula**

| **Antipsychotic** | **Posology** | **No. Adm./Y** | **Posology Score (PoS)** | **Release mode** | **Release Score (RS)** | **Administration Index (AI)** | **Special requests** | **Special requests index (SRI)** | **CYP (induced/inhibited)** | **CYP Index (CYPI)** | **CAS (%)** |
| --- | --- | --- | --- | --- | --- | --- | --- | --- | --- | --- | --- |
| Chlorpromazine | p.o. BID | 730,5 | -1,00 | immediate release | 0 | -0,500 | No | 1 | None | 1 | 50,00% |
| Chlorpromazine | p.o. TID | 1095,75 | -2,00 | immediate release | 0 | -1,000 | No | 1 | None | 1 | 33,33% |
| Chlorpromazine | p.o. QID | 1461 | -3,00 | immediate release | 0 | -1,500 | No | 1 | None | 1 | 16,67% |
| Flupenthixol | LAI 2W | 24 | 0,93 | long acting | 1 | 0,967 | No | 1 | None | 1 | 98,90% |
| Flupenthixol | LAI 1M | 12 | 0,97 | long acting | 1 | 0,984 | No | 1 | None | 1 | 99,45% |
| Fluphenazine | p.o. QID | 1461 | -3,00 | immediate release | 0 | -1,500 | No | 1 | None | 1 | 16,67% |
| Fluphenazine | p.o. TID | 1095,75 | -2,00 | immediate release | 0 | -1,000 | No | 1 | None | 1 | 33,33% |
| Fluphenazine | LAI 1M | 12 | 0,97 | long acting | 1 | 0,984 | No | 1 | None | 1 | 99,45% |
| Fluphenazine | LAI 6W | 9 | 0,98 | long acting | 1 | 0,988 | No | 1 | None | 1 | 99,59% |
| Haloperidol | p.o. BID | 730,5 | -1,00 | immediate release | 0 | -0,500 | No | 1 | CYP2D6 (inhibitor) | 0 | 16,67% |
| Haloperidol | p.o. TID | 1095,75 | -2,00 | immediate release | 0 | -1,000 | No | 1 | CYP2D6 (inhibitor) | 0 | 0,00% |
| Haloperidol | LAI 1M | 12 | 0,97 | long acting | 1 | 0,984 | No | 1 | CYP2D6 (inhibitor) | 0 | 66,12% |
| Loxapine | p.o. BID | 730,5 | -1,00 | immediate release | 0 | -0,500 | No | 1 | None | 1 | 50,00% |
| Loxapine | p.o. TID | 1095,75 | -2,00 | immediate release | 0 | -1,000 | No | 1 | None | 1 | 33,33% |
| Loxapine | p.o. QID | 1461 | -3,00 | immediate release | 0 | -1,500 | No | 1 | None | 1 | 16,67% |
| Levomepromazine (Methotrimeprazine) | p.o. TID | 1095,75 | -2,00 | immediate release | 0 | -1,000 | No | 1 | None | 1 | 33,33% |
| Periciazine | p.o. BID | 730,5 | -1,00 | immediate release | 0 | -0,500 | No | 1 | CYP2D6 (inhibitor) | 0 | 16,67% |
| Perphenazine | p.o. QID | 1461 | -3,00 | immediate release | 0 | -1,500 | No | 1 | CYP2D6 (inhibitor) | 0 | -16,67% |
| Perphenazine | p.o. TID | 1095,75 | -2,00 | immediate release | 0 | -1,000 | No | 1 | CYP2D6 (inhibitor) | 0 | 0,00% |
| Perphenazine | p.o. BID | 730,5 | -1,00 | immediate release | 0 | -0,500 | No | 1 | CYP2D6 (inhibitor) | 0 | 16,67% |
| Pimozide | p.o. QD | 365,25 | 0,00 | immediate release | 0 | 0,000 | No | 1 | None | 1 | 66,67% |
| Thioridazine | p.o. QID | 1461 | -3,00 | immediate release | 0 | -1,500 | No | 1 | CYP2D6 (inhibitor) | 0 | -16,67% |
| Thioridazine | p.o. TID | 1095,75 | -2,00 | immediate release | 0 | -1,000 | No | 1 | CYP2D6 (inhibitor) | 0 | 0,00% |
| Thioridazine | p.o. BID | 730,5 | -1,00 | immediate release | 0 | -0,500 | No | 1 | CYP2D6 (inhibitor) | 0 | 16,67% |
| Thiothixene | p.o. BID | 730,5 | -1,00 | immediate release | 0 | -0,500 | No | 1 | None | 1 | 50,00% |
| Thiothixene | p.o. TID | 1095,75 | -2,00 | immediate release | 0 | -1,000 | No | 1 | None | 1 | 33,33% |
| Trifluoperazine | p.o. BID | 730,5 | -1,00 | immediate release | 0 | -0,500 | No | 1 | None | 1 | 50,00% |
| Zuclopenthixol | p.o. QD | 365,25 | 0,00 | immediate release | 0 | 0,000 | No | 1 | None | 1 | 66,67% |
| Zuclopenthixol | LAI 1M | 12 | 0,97 | long acting | 1 | 0,984 | No | 1 | None | 1 | 99,45% |
| Asenapine | p.o. BID | 730,5 | -1,00 | immediate release | 0 | -0,500 | No | 1 | CYP2D6 (inhibitor) | 0 | 16,67% |
| Clozapine | p.o. QD | 365,25 | 0,00 | immediate release | 0 | 0,000 | Yes | 0 | None | 1 | 33,33% |
| Clozapine | p.o. BID | 730,5 | -1,00 | immediate release | 0 | -0,500 | Yes | 0 | None | 1 | 16,67% |
| Iloperidone | p.o. BID | 730,5 | -1,00 | immediate release | 0 | -0,500 | No | 1 | None | 1 | 50,00% |
| Sertindole | p.o. QD | 365,25 | 0,00 | immediate release | 0 | 0,000 | Yes | 0 | None | 1 | 33,33% |
| Lumateperone | p.o. QD | 365,25 | 0,00 | immediate release | 0 | 0,000 | No | 1 | None | 1 | 66,67% |
| Lurasidone | p.o. QD | 365,25 | 0,00 | immediate release | 0 | 0,000 | No | 1 | None | 1 | 66,67% |
| Olanzapine | p.o. QD | 365,25 | 0,00 | immediate release | 0 | 0,000 | No | 1 | None | 1 | 66,67% |
| Olanzapine | LAI 2W | 24 | 0,93 | long acting | 1 | 0,967 | Yes | 0 | None | 1 | 65,57% |
| Olanzapine | LAI 1M | 12 | 0,97 | long acting | 1 | 0,984 | Yes | 0 | None | 1 | 66,12% |
| Zotepine | p.o. TID | 1095,75 | -2,00 | immediate release | 0 | -1,000 | No | 1 | None | 1 | 33,33% |
| Paliperidone | p.o. QD | 365,25 | 0,00 | extended release | 0,5 | 0,250 | No | 1 | None | 1 | 75,00% |
| Paliperidone | LAI 1M | 12 | 0,97 | long acting | 1 | 0,984 | No | 1 | None | 1 | 99,45% |
| Paliperidone | LAI 3M | 4 | 0,99 | long acting | 1 | 0,995 | No | 1 | None | 1 | 99,82% |
| Paliperidone | LAI 6M | 2 | 0,99 | long acting | 1 | 0,997 | No | 1 | None | 1 | 99,91% |
| Quetiapine | p.o. BID | 730,5 | -1,00 | immediate release | 0 | -0,500 | No | 1 | None | 1 | 50,00% |
| Quetiapine | p.o. QD | 365,25 | 0,00 | extended release | 0,5 | 0,250 | No | 1 | None | 1 | 75,00% |
| Amisulpride | p.o. QD | 365,25 | 0,00 | immediate release | 0 | 0,000 | No | 1 | None | 1 | 66,67% |
| Amisulpride | p.o. BID | 730,5 | -1,00 | immediate release | 0 | -0,500 | No | 1 | None | 1 | 50,00% |
| Risperidone | p.o. QD | 365,25 | 0,00 | immediate release | 0 | 0,000 | No | 1 | None | 1 | 66,67% |
| Risperidone | LAI 2W | 24 | 0,93 | long acting | 1 | 0,967 | No | 1 | None | 1 | 98,90% |
| Risperidone | LAI 1M | 12 | 0,97 | long acting | 1 | 0,984 | No | 1 | None | 1 | 99,45% |
| Ziprasidone | p.o. BID | 730,5 | -1,00 | immediate release | 0 | -0,500 | No | 1 | None | 1 | 50,00% |
| Aripiprazole^(a)^ | p.o. QD | 365,25 | 0,00 | immediate release | 0 | 0,000 | No | 1 | None | 1 | 66,67% |
| Aripiprazole^(a)^ | LAI 1M | 12 | 0,97 | long acting | 1 | 0,984 | No | 1 | None | 1 | 99,45% |
| Aripiprazole^(a)^ | LAI 6W | 9 | 0,98 | long acting | 1 | 0,988 | No | 1 | None | 1 | 99,59% |
| Aripiprazole^(a)^ | LAI 2M | 6 | 0,98 | long acting | 1 | 0,992 | No | 1 | None | 1 | 99,73% |
| Brexpiprazole^(a)^ | p.o. QD | 365,25 | 0,00 | immediate release | 0 | 0,000 | No | 1 | None | 1 | 66,67% |
| Cariprazine^(a)^ | p.o. QD | 365,25 | 0,00 | immediate release | 0 | 0,000 | No | 1 | None | 1 | 66,67% |
| Theoretical maximum | ideal* | 0 | 1,00 | ideal | 1 | 1,000 | No | 1 | None | 1 | 100,00% |
